# Robustness of the Quadratic Discriminant Function to correlated and uncorrelated normal training samples

**DOI:** 10.1186/s40064-016-1718-3

**Published:** 2016-02-01

**Authors:** Atinuke Adebanji, Michael Asamoah-Boaheng, Olivia Osei-Tutu

**Affiliations:** Department of Mathematics, Kwame Nkrumah University of Science and Technology, PMB KNUST, Kumasi, Ghana; Institute of Research, Innovation and Development (IRID), Kumasi Polytechnic, Box 854, Kumasi, Ghana

**Keywords:** QDF, Correlated normal, Uncorrelated normal, Group centroid

## Abstract

This study investigates the asymptotic performance of the Quadratic Discriminant Function (QDF) under correlated and uncorrelated normal training samples. This paper specifically examines the effect of correlation, uncorrelation considering different sample size ratios, number of variables and varying group centroid separators ($\delta$, $\delta = 1; 2; 3; 4; 5$) on classification accuracy of the QDF using simulated data from three populations ($\pi _{i}, i=1,2,3$). The three populations differs with respect to their mean vector and covariance matrices. The results show the correlated normal distribution exhibits high coefficient of variation as $\delta$ increased. The QDF performed better when the training samples were correlated than when they were under uncorrelated normal distribution. The QDF performed better resulting in the reduction in misclassification error rates as group centroid separator increases with non increasing sample size under correlated training samples.

## Background

Discriminant analysis (DA) as a topic in Multivariate Statistical Analysis has attracted much research interest over the years, with the evaluation of discriminant functions when the covariances matrices are unequal with moderate sizes being well explained by Wahl and Kronmal (1977). Linear discriminant function (LDF) is commonly used by researchers because of its simplicity of form and concept. In spite of theoretical evidence supporting the use of the Quadratic Discriminant Function (QDF) when the covariance matrices are heterogeneous, its actual employment has been sporadic because there are unanswered questions regarding its performance in the practical situation where the discriminant function must be constructed using training samples that do not satisfy the classical assumption of the model. The pioneering work on quadratic discrimination was by Smith (1947) using Fisher’s Iris data. He provided a full expression for the QDF and his results showed the QDF outperforming the LDF when the homogeneity of variance covariance structure was violated.

Marks and Dunn (1974) approached the problem of discrimination by comparing the asymptotic and small sample performance of the QDF, best linear and Fisher’s LDF for both proportional and non-proportional covariance differences under the assumption of normality and unequal covariance matrices. Two populations were used and sample sizes were chosen from 10 to 100. The number of variables selected were 2 and 10. They employed the application of Monte Carlo simulation. Their results indicated that for small samples the QDF performed worse than the LDF when covariances were nearly equal with large dimensions (ie LDF was satisfactory when the covariance matrices were not too different).

Lawoko (1988) studied the performance of the LDF and QDF under the assumption of correlated training samples. The researcher aimed at allocating an object to one of two groups on the basis of measurements on the object. He found that the discriminant functions formed under the model did not perform better than *W* and *Z* formed under the assumption of independent training observation. Asymptotic expected error rate for *W* under the model ($W_m$) and *W* were equal when the training observations followed an autoregressive process but there was a slight improvement in the overall error rate when $W_m$ was used instead of *W* for numerical evaluations of the asymptotic expansions. He concluded that the efficiency of the discriminant analysis estimator is generally lowered by positively correlated training observations. Mardia et al. (1995) reported that it might be thought that a linear combination of two variables would provide a better discriminator if they were correlated than when they were uncorrelated. However, this is not necessarily so. To show this they considered two bivariate populations $\pi _{1}$ and $\pi _{2}$. Supposing $\pi _{1}$ is $N_{2}(0,\Sigma )$ and $\pi _{2}$ is $N_{2}(\mu ,\Sigma )$ where $\mu =(\mu _{1},\mu _{2})'$ and with known $\Sigma$. They indicated that discrimination is improved unless $\rho$ lies between zero and $2f/{(1 + f^{2}})$ but a small value of $\rho$ can actually harm discrimination.

Adebanji and Nokoe (2004) have considered evaluating the quadratic classifier. They restricted their attention to two multivariate normal populations of independent variables. In addition to some theoretical result, with known parameters, they conducted a Monte Carlo simulation in order to investigate the error rates. Results indicated that the total error rate computed showed that there was an increase in the error rate with re-substitution estimator for all *K* values. On the other hand, there was a decline across *K*. The cross-validation estimator showed a steady decline for and across all values *K* and the recorded value showed a substantially low error rate estimates than re-substitution estimator for $K=4$ and $K=8$.

Kakaï and Pelz (2010) studied the asymptotic error rates of linear, quadratic rules and conducted a Monte Carlo study in 2, 3 and 5-group discriminant analysis. Hyodo and Kubokawa (2014) studied a variable selection criterion for linear discriminant rule and its optimality in high dimensional data where a new variable procedure was developed for selecting the true variable set.

An enormous deal of study has been made since Fisher’s (1936) original work on discriminant analysis as well as several other researchers tackling similar problem. Some estimation methods have been proposed and some sampling properties derived. However, there is little investigation done on large sample properties of these functions. Also a considerable number of studies had been carried out on discriminant analysis but not much is done on the effect or the performance of the QDF under correlated and uncorrelated data with varying sample size ratios, different variable selections and with different centroid separators for three populations.

In this study we therefore investigate the performance of classification functions (i.e Quadratic Discriminant Functions) when the covariance matrices are heterogeneous with the data of interest being correlated, sample size ratios being unequal, considering different number of variables and varying values of group centroid separator ($\delta$).

## Methods

### Simulation design

To evaluate the performance of QDF for correlated and uncorrelated training samples of distributions, we considered a Monte Carlo study with multivariate normally correlated random data generated for three populations with their mean vector $$\mu _1=(0, \ldots , 0)$$, $$\mu _2=(0, \ldots , \delta )$$ and $$\mu _3=(0, \ldots , 2\delta )$$ respectively. The covariance matrices, $$\Sigma _i$$ (i = 1, 2, 3). Where $$k\ne l,$$$$\sigma _{kl}=0.7$$ for all groups except the diagonal entries given as $$\sigma _{k}^2 =i$$, for $$i=1,2,3$$. The covariance matrices were transformed to be uncorrelated to generate the uncorrelated data. The QDF was then performed in each case and the leave-one-out method was used to estimate the proportion of observations misclassified.

Factors considered in this study were:

1. Mean vector separator which is set at $\delta$ from 1 to 5 where $\delta$ is determined by the difference between the mean vectors.
2. Sample sizes which are also specified. Here 14 values of $n_1$ set at 30, 60, 100, 150, 200, 250, 300, 400, 500, 600, 700, 800, 1000, 2000 and the sample size of $n_2$ and $n_3$ are determined by the sample ratios at 1:1:1, 1:2:2 and 1:2:3 and these ratios also determine the prior probabilities to be considered.
3. The number of variables for this study is also specified. The number of variables are set at 4, 6 and 8 following Murray (1977) who considered this in selection of variables in discriminant analysis.
4. The size of population 1 $(n_1)$ is fixed throughout the study and the sizes of population 2 and population 3, $n_2$ and $n_3$ respectively are determined by the sample size ratio under consideration.

### Subroutine for QDF

Series of subroutines were written in MatLab to perform the simulation and discrimination procedures on QDF. Below are the important ones.

### Classification into several populations

Generalization of classification procedure for more than two discriminating groups (ie from 2 to $g\ge 2$) is straight forward. However, not much is known about the properties corresponding sample classification function, and in particular, their error rates have not been fully investigated. Therefore, we focus only on the Minimum ECM classification with equal misclassification cost and Minimum TPM for multivariate normal population with unequal covariance matrices (quadratic discriminant analysis).

#### Minimum ECM classification with equal misclassification cost

Allocate ${\mathbf {x}}_0$ to $\Pi _k$ if

1 $$\begin{aligned} p_k f_k({\mathbf {x}})> p_i f_i({\mathbf {x}}) \quad \text {for all}\; i\ne k \end{aligned}$$

or, equivalently, Allocate ${\mathbf {x}}_0$ to $\Pi _k$ if

2 $$\begin{aligned} \ln p_k f_k({\mathbf {x}})> \ln p_i f_i({\mathbf {x}}) \quad \text {for all}\;i\ne k \end{aligned}$$

Note that the classification rule in Eq. (1) is identical to the one that maximizes the posterior probability $P(\Pi _i|{\mathbf {x}})=$ P(${\mathbf {x}}$ comes from $\Pi _i$ given that ${\mathbf {x}}$ is observed) where

3 $$\begin{aligned} P(\Pi _i|{\mathbf {x}})=\frac{p_k f_k({\mathbf {x}})}{\sum _{i=1}^{g}p_i f_i({\mathbf {x}})}= \frac{(prior)\times (likelihood)}{\sum [(prior)\times (likelihood)]} \end{aligned}$$

Therefore, one should keep in mind that in general minimum ECM rule must have the prior probability, misclassification cost and density function before it can be implemented.

#### Minimum TPM rule for unequal-covariance normal populations

Suppose that the $\Pi _i$ are multivariate normal populations, with different mean vectors ${\mathbf {\mu }}$ and covariance matrices ${\Sigma }_i \, (i=1,\ldots ,g)$. An important special case occurs when the

$$\begin{aligned} f_i({\mathbf {x}})=\frac{1}{(2\pi )^{p/2}|{\Sigma }_i|^{\frac{1}{2}}} \exp \left\{ -\frac{1}{2}({\mathbf {x}}-{\mathbf {\mu }}_i)'{\Sigma }_{i}^{-1} ({\mathbf {x}}-{\mathbf {\mu }}_i)\right\} \end{aligned}$$

with $$c(i\mid i)=0$$, $$c(k\mid i)=1, k\ne i$$ then

4 $$\begin{aligned} \ln p_k f_k({\mathbf {x}})= & {} \ln {p_k}-\left( \frac{p}{2}\right) \ln \{(2\pi )\}-\frac{1}{2}\ln |{\Sigma }_k|- \frac{1}{2}({\mathbf {x}}-{\mathbf {\mu }}_k)'{\Sigma }_{k}^{-1}({\mathbf {x}} -{\mathbf {\mu }}_k)\nonumber \\= & {} \max _i\ln \{p_i f_i({\mathbf {x}})\} \end{aligned}$$

The constant $(p/2)\ln (2\pi )$ can be ignored in Eq. (4), since it is the same for all population. Therefore, quadratic discriminant score for *ith* population is defined as

5 $$\begin{aligned} d_{i}^{Q}({\mathbf {x}})=-\frac{1}{2}\ln |{\Sigma }_i|-\frac{1}{2}({\mathbf {x}} -{\mathbf {\mu }}_i)'{\Sigma }_{i}^{-1}({\mathbf {x}}-{\mathbf {\mu }}_i)+ \ln {p_i} \end{aligned}$$

The quadratic score $d_{i}^{Q}({\mathbf {x}})$ is composed of contributions from the generalized variance $|{\Sigma }_i|$, the prior probability $p_i$, and the square of the distance from *x* to the population mean $\mu _i$.

Allocate *x* to $\Pi _k$ if the quadratic score

6 $$\begin{aligned} d_{k}^{Q}({\mathbf {x}})= \text {largest of}\ d_{1}^{Q}({\mathbf {x}}), d_{2}^{Q}({\mathbf {x}}),\ldots , d_{g}^{Q}({\mathbf {x}}). \end{aligned}$$

In practice, the $\mu _i$ and $\Sigma _i$ are unknown, but a training set of correctly classified observations if often available for the construction of estimates. The relevant sample quantities for population $\Pi _i$ are the sample mean vector, $\bar{x}_i$, sample covariance matrix, $S_i$ and sample size, $n_i$. The estimate of the quadratic discriminant score (6) is then

7 $$\begin{aligned} \hat{d}_{i}^{Q}({\mathbf {x}})=-\frac{1}{2}\ln |{\mathbf {S}}_i|-\frac{1}{2} ({\mathbf {x}}-{\bar{\mathbf {x}}}_i)'{\mathbf {S}}_{i}^{-1}({\mathbf {x}}-{\bar{\mathbf {x}}}_i)+ \ln {p_i} \quad \text {for}\quad i=1, 2,\ldots ,g \end{aligned}$$

### The quadratic classifier ($\Sigma _1\ne \Sigma _2$)

Suppose that the joint densities of $X'=[X_1,X_2,\ldots ,X_p] \text{ for } \text{ population } \Pi _1$ and $\Pi _2$ are given by

8 $$\begin{aligned} f_i({\mathbf {x}})=\frac{1}{\left( 2\pi \right) ^{p/2}\left| \Sigma _{i}\right| ^{1/2}}exp \left[ -\frac{1}{2}({\mathbf {x}}-\mu _{i})'\Sigma _{i}^{-1}({\mathbf {x}}-\mu _{i})\right] \end{aligned}$$

The covariance matrices as well as the mean vectors are different from one another for the two populations. The regions of minimum expected cost misclassification (ECM) and minimum total probability of misclassification (TPM) depends on the ratio of the densities, $(f_1({\mathbf {x}}))/(f_2({\mathbf {x}})$, or equivalently, the natural logarithm of the density ratio, $\ln [(f_1({\mathbf {x}})/(f_2({\mathbf {x}})]=\ln [f_1({\mathbf {x}})]-\ln [f_2({\mathbf {x}})]$ when the multivariate normal densities have different covariance structures, the terms in the density ratio involving $\left| \Sigma _{i}^{1/2}\right|$ do not cancel as they do when we have equal covariance matrices and also the quadratic forms in the exponents of $f_i({\mathbf {x}})$ do not combine. Therefore substituting multivariate normal densities with different covariance matrices into Eq. (1) and after taking the natural logarithms and simplifying, the likelihood of the density ratios gives the quadratic function in ${\mathbf {x}}\in \Pi _1$ if

$$\begin{aligned} -\frac{1}{2}{{\mathbf {x}}'}(\Sigma ^{-1}_{1}-\Sigma ^{-1}_{2}){\mathbf {x}} +(\mu '_{1}\Sigma ^{-1}_{1}-\mu '_{2}\Sigma ^{-1}_{2}){\mathbf {x}}-k\ge \ln \left[ {\left( \frac{c(1|2)}{c(2|1)}\right) \left( \frac{p_2}{p_1}\right) }\right] , \end{aligned}$$

where

9 $$\begin{aligned} k=\frac{1}{2}\ln \left( \frac{|\Sigma _1|}{|\Sigma _2|}\right) +\frac{1}{2}(\mu '_{1}\Sigma ^{-1}_{1}\mu _{1}-\mu '_{2}\Sigma ^{-1}_{2}\mu _{2}) \end{aligned}$$

otherwise, $${\mathbf {x}}\in \Pi _1$$.

This function is easily extended to the 3 group classification where 2 cut off points are required for assigning observations to the 3 groups (Johnson and Wichern 2007).

## Results

This section presents the performance of QDF when the training data are correlated and then when they are uncorrelated.

### Effects of sample size on QDF under correlated and uncorrelated normal distribution

**Fig. 1:**
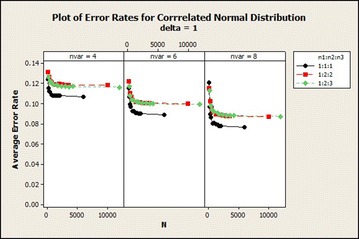
Average error rates of correlated normal distribution for $\delta$: $n_1{:}n_2{:}n_3=1{:}1{:}1$

Evaluating the effect of sample size on QDF with respect to the correlated normal distribution for $\delta = 1$ is present in Fig. 1. From Fig. 1 it was observed that the average error rate for 4, 6 and 8 variables with $\delta =1$ were higher as compared to the other values of the $\delta$ and among the sample size ratios used, sample size ratio 1:1:1 gives the lowest average error rates as the sample size increases asymptotically. Results also show that $n_1=30$ gave highest average error rates and lower average error rate are for $n_1=2000$ for variables 4, 6 and 8. There is a rapid decrease in the average error rate from total sample size of 90–180 of sample size ratio (1:1:1) of 8 variables for all $\delta$. The results of 4 variables were higher than the other number of variables. $\delta =5$ gave the lowest average error rates as the sample size increases. It was also observed that the average error rates of sample size ratio 1:1:1 and 1:2:2 were marginal for $\delta =1$. The difference between the ratios decreased as $\delta$ increased and with maintained total sample size and the average error rates decreased as the number of variables increased. In $\delta =5$ the performance of the three sample size ratios were marginal.

**Table 1:** Effects of sample size on QDF for correlated normal based on error rates, CV and SD

Centroid	Sample size (n)	SD	CV	Mean error rate
$\delta = 1$	90	0.0530	0.04276	0.1240
	180	0.0536	0.04656	0.1151
	300	0.0543	0.04853	0.1120
	450	0.0547	0.04903	0.1116
	750	0.0534	0.04908	0.1089
	900	0.0526	0.04857	0.1082
	1200	0.0524	0.04868	0.1077
	1500	0.0529	0.04893	0.1081
	1800	0.0526	0.04881	0.1077
	2100	0.0531	0.04913	0.1080
	6000	0.0524	0.04898	0.1069
$\delta = 2$	90	0.0447	0.05084	0.0880
	180	0.0402	0.05215	0.0771
	300	0.0384	0.05224	0.0735
	450	0.0394	0.05439	0.0724
	750	0.0370	0.05172	0.0715
	900	0.0370	0.05217	0.0710
	1200	0.0369	0.05249	0.0703
	1500	0.0367	0.05191	0.0707
	1800	0.0372	0.05265	0.0706
	2100	0.0367	0.05222	0.0702
	6000	0.0365	0.05234	0.0698
$\delta = 3$	90	0.0300	0.6047	0.0479
	180	0.0264	0.5983	0.0442
	300	0.0245	0.5940	0.0412
	450	0.0239	0.5907	0.0495
	750	0.0232	0.5780	0.0401
	900	0.0230	0.5763	0.0400
	1200	0.0226	0.5708	0.0396
	1500	0.0225	0.5727	0.0393
	1800	0.0223	0.5682	0.0393
	2100	0.0226	0.5728	0.0395
	6000	0.0224	0.5698	0.0393
$\delta = 4$	90	0.0200	0.7509	0.0266
	180	0.0159	0.7195	0.0221
	300	0.0144	0.6727	0.0215
	450	0.0139	0.6732	0.0207
	750	0.0135	0.6574	0.0205
	900	0.0131	0.6455	0.0203
	1200	0.0124	0.6286	0.0198
	1500	0.0126	0.6353	0.0199
	1800	0.0128	0.6301	0.0203
	2100	0.0128	0.6382	0.0201
	6000	0.0125	0.6284	0.0200
$\delta = 5$	90	0.0123	0.9798	0.0126
	180	0.0094	0.8717	0.0108
	300	0.0084	0.8252	0.0102
	450	0.0078	0.7838	0.0100
	750	0.0071	0.7489	0.0095
	900	0.0069	0.7399	0.0093
	1200	0.0070	0.7158	0.0098
	1500	0.0066	0.7058	0.0093
	1800	0.0066	0.7016	0.0093
	2100	0.0065	0.7002	0.0093
	6000	0.0064	0.6843	0.0094

From Table 1, the effects of the sample size on the QDF for the various group centroids ($\delta =1,2,3,4,5$) for the correlated samples gave an indication that, generally as the sample size increases with increasing group centroids, the mean error rates decreases marginally in that order. The standard deviation of the error rate for the correlated normal distribution reveals that as the sample size increases, standard deviation of the error rate for sample size ratio 1:1:1 exhibit low standard deviations for $\delta =1$. For a particular $\delta$, the standard deviation decreases as the number of variables also increases. From $\delta =2$ to $\delta =5$, the standard deviations decreases as the sample size increases asymptotically. There is a sharp decrease of the standard deviation of sample size ratio 1:1:1.

**Fig. 2:**
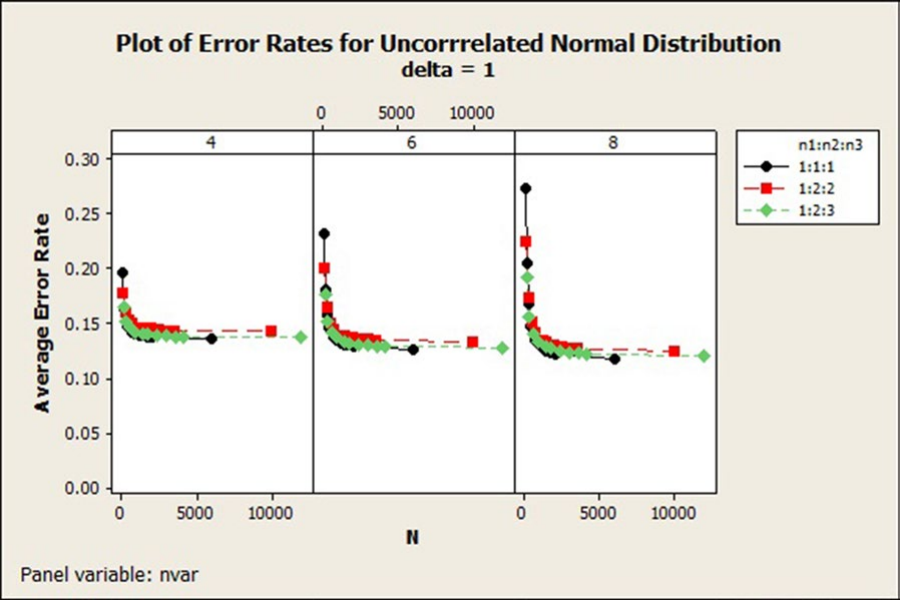
Average error rates of uncorrelated normal distribution: $\delta =1$

For the uncorrelated distribution from Table 2 the average error rate was similar to the results obtained in the correlated normal distribution with the exception of the average error rate of sample size ratio 1:1:1 which decreased rapidly from total sample size of 90–180 for 8 variables in all $\delta$s. The average error rate decreased as the total sample size increased asymptotically. And it reduced when $\delta$ also increased. The graphical representation of this result for $\delta =1$ is shown in Fig. 2.

**Table 2:** Effects of sample size on QDF for uncorrelated normal based on error rates, CV and SD

Centroid	Sample size (n)	SD	CV	Mean error rate
$\delta = 1$	90	0.0681	0.3483	0.1955
	180	0.0641	0.3920	0.1636
	300	0.0603	0.3965	0.1522
	450	0.0603	0.4092	0.1473
	750	0.0575	0.4059	0.1417
	900	0.0573	0.4065	0.1409
	1200	0.1387	0.4096	0.1387
	1500	0.0571	0.4114	0.1388
	1800	0.0567	0.4123	0.1374
	2100	0.0567	0.4129	0.1374
	6000	0.0559	0.4116	0.1359
$\delta = 2$	90	0.0524	0.3968	0.1321
	180	0.0469	0.4222	0.1111
	300	0.0421	0.4062	0.1037
	450	0.0424	0.4251	0.0997
	750	0.0409	0.4218	0.0970
	900	0.0415	0.4313	0.0962
	1200	0.0402	0.4209	0.0955
	1500	0.0398	0.4219	0.0944
	1800	0.0400	0.4230	0.0946
	2100	0.0399	0.4239	0.0940
	6000	0.0394	0.4223	0.0934
$\delta = 3$	90	0.0345	0.4200	0.0823
	180	0.0296	0.4341	0.0683
	300	0.0277	0.4417	0.0028
	450	0.0263	0.4395	0.0599
	750	0.0256	0.4372	0.0586
	900	0.0257	0.4385	0.0586
	1200	0.0255	0.4393	0.0581
	1500	0.0253	0.4386	0.0578
	1800	0.0248	0.4330	0.0573
	2100	0.0250	0.4361	0.0574
	6000	0.0249	0.4380	0.0568
$\delta = 4$	90	0.0236	0.4866	0.0486
	180	0.0189	0.4991	0.0379
	300	0.0173	0.4889	0.0354
	450	0.0160	0.4802	0.0334
	750	0.0158	0.4815	0.0327
	900	0.0153	0.4744	0.0323
	1200	0.0151	0.4725	0.0319
	1500	0.0151	0.4728	0.0318
	1800	0.0147	0.4660	0.0315
	2100	0.0147	0.4663	0.0315
	6000	0.0146	0.4656	0.0313
$\delta = 5$	90	0.0156	0.5989	0.0260
	180	0.0121	0.5892	0.0205
	300	0.0098	0.5613	0.0174
	450	0.0093	0.5367	0.0173
	750	0.0090	0.5507	0.0163
	900	0.0085	0.5262	0.0161
	1200	0.0085	0.5260	0.0162
	1500	0.0085	0.5272	0.0161
	1800	0.0083	0.5182	0.0160
	2100	0.0083	0.5221	0.0159
	6000	0.0081	0.5109	0.0158

**Fig. 3:**
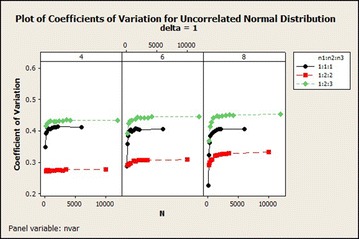
Coefficients of variation for uncorrelated normal distribution: $\delta =1$

The coefficients of variation generally increased exponentially and stabilized with increasing total sample size and number of variables in $\delta =1$ exhibited lower variations as compared with the remaining $\delta$s as shown in Fig. 3. For $\delta =4$, the coefficients of variation in sample size ratio 1:1:1 decreased while the remaining ratios did not give any particular pattern for the 4 variable situation. For 4 variable situation with $\delta =5$, the coefficients of variation decreased as the total sample size increased. The coefficient of variation of the other 6 and 8 variables situations did not show any particular pattern as the total sample size increased.

**Fig. 4:**
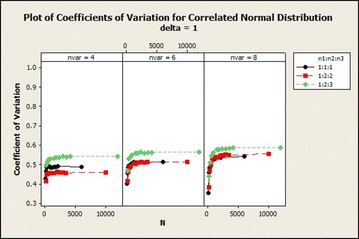
Coefficients of variation for correlated normal distribution: $\delta =1$

The coefficients of variation in correlated normal distribution in Fig. 2 increased exponentially and then stabilized with averagely lower variations in sample size ratio 1:2:2 and with higher variations in sample size ratio 1:2:3 as the total sample size increases asymptotically. The variations also increased as $\delta$ increased. $\delta =3$ gives a steady coefficients of variation as the total sample size increased for variable 4 while it gave a little increase and then stabilized in variables 4 and 6. There was a decline in the coefficients of variation for $\delta =4$ as the total sample size increased asymptotically in variable 4. The coefficients of variation increased from total sample size 150 to 500 and from 180 to 360 for sample size ratios 1:2:2 and 1:2:3 respectively for variables 6 and 8 and then decreased as the total sample size increased asymptotically. From Fig. 4, for $\delta =1$, there was a sharp decrease in the coefficients of variation in sample size ratio 1:1:1 for all number of variables as the total sample size increased.

### Effect of number of variables on QDF (under correlated and uncorrelated normal distribution)

The effect of number of variables on the QDF under the correlated and uncorrelated normal distribution are discussed under this subsection.

**Fig. 5:**
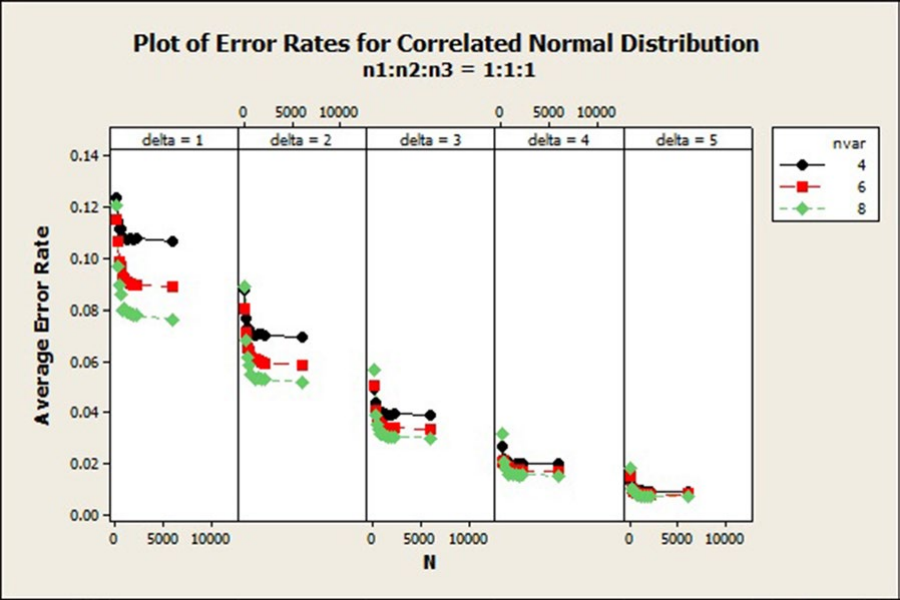
Average error rate for correlated normal distribution: $n_1{:}n_2{:}n_3=1{:}1{:}1$

The graphs of the results for sample size ratio 1:1:1 of the situations of 4, 6 and 8 variables are shown in Fig. 5. It was observed that as the number of variables increased, the average error rate reduced in the correlated normal distribution. The rate at which it reduces in $\delta =1$ for ratio 1:1:1 is better than that of the other $\delta$s. For increasing sample size ratio, as the number of variables increased, the decrease in the average error was marginal as $\delta$ increased.

**Fig. 6:**
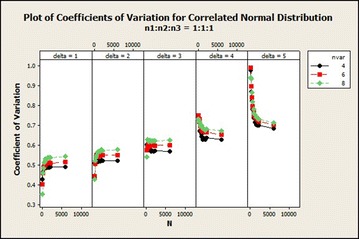
Coefficient of variation of correlated normal distribution: $n_1{:}n_2{:}n_3=1{:}1{:}1$

The coefficients of variation in this distribution for ratio 1:1:1 in Fig. 6 reveals that as the number of variables increased the coefficients of variation increased for variables 4, 6 and 8 from $\delta =1$ to 3 except $\delta =4$ and 5 in which it reduced. Yet the in the case of 8 variables the variabilities exhibited were higher than the rest in this case. For ratio 1:2:2 the coefficients of variation increased from total sample size of 150–2000 and stabilized for all $\delta$s as the number of variables increased except $\delta =4$ which showed a decline in the coefficients of variation for the case of 4 and 6 variables. In $\delta =5$, there was declination in the coefficients of variation as the number of variables increased. Sample size ratio 1:2:3 gave similar result as ratio 1:2:2

**Fig. 7:**
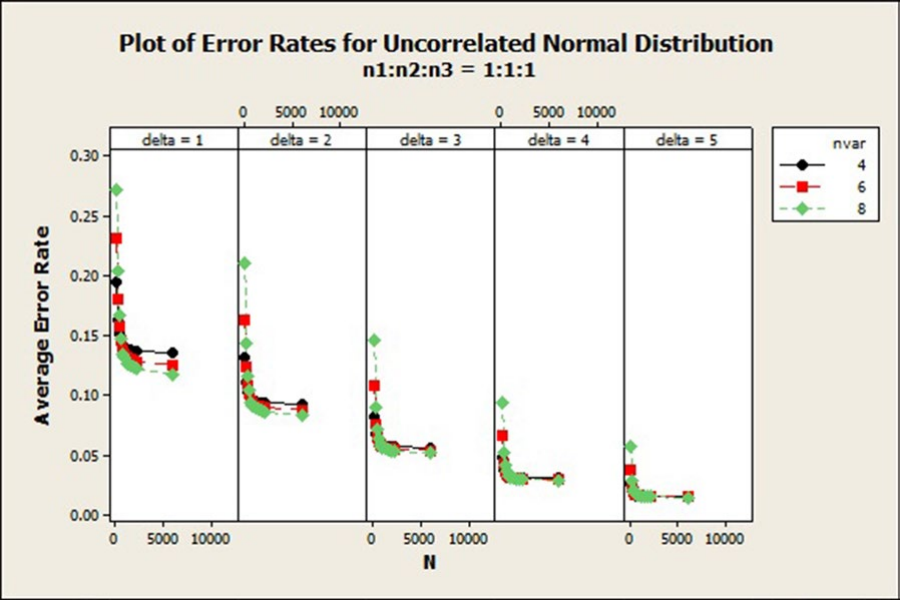
Average error rates of uncorrelated normal distribution: $n_1{:}n_2{:}n_3=1{:}1{:}1$

From Fig. 7, there was a sharp decline in the average error rate from total sample size 90–180 as the number of variables increase for all $\delta$s. It also revealed that as the number of variables increased the average error rate reduced for all sample size ratios.

**Fig. 8:**
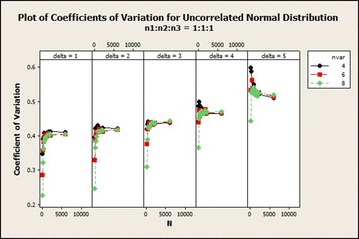
Coefficients of variation for uncorrelated normal distribution: $n_1{:}n_2{:}n_3=1{:}1{:}1$

The coefficients of variation shown in Fig. 8 indicates that the variabilities increased exponentially for all $\delta$s with the exception of $\delta =4$ and 5 for which variable 4 declined. In the case of 8 variables, about 9.65 and 11.91 % increase in variations from total sample size of 90–180 for all $\delta =1$ and 2. For $\delta =4$ and 5, the coefficients of variation for variables 4 declined from total sample size of 90–6000 while variables 6 and 8 increased. For $\delta =5$, the coefficients of variations for 8 variables increased from 90 to 750 and declined to 6000. The coefficients of variation in general for this distribution increased as the number of variables increased.

### Effect of group centroid separator on QDF under correlated and uncorrelated normal distribution

**Fig. 9:**
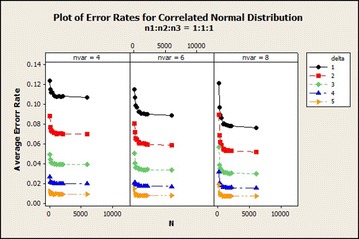
Average error rates of correlated normal distribution for $\delta$: $n_1{:}n_2{:}n_3=1{:}1{:}1$

This section presents the results of our investigation on the effect of the Mahalanobis distance on QDF for correlated normal distribution. Considering the correlated normal distribution in Fig. 9, it was observed that with increasing total sample size, the average error rate reduces as the $\delta$ increased and also reduced as the number of variables increased. It can be observed that there was about 2.37 % drop in the average error rate from total sample size 90–180 for all $\delta =1$s in the case of 8 variables. The average error rate reduced as the total sample size increased for all sample size ratios with increasing $\delta$.

**Fig. 10:**
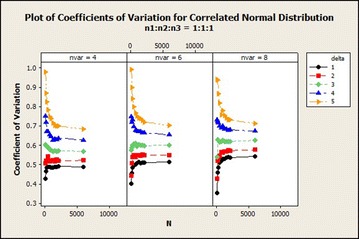
Coefficients of variation for correlated normal distribution: $n_1{:}n_2{:}n_3=1{:}1{:}1$

The coefficients of variation of sample size ratio 1:1:1 with increasing total sample size in Fig. 10, uniform behaviour of $\delta$ was not portrayed. As coefficients of variation for $\delta =5$ and 4 were declining, that of the rest of the $\delta$s may be increasing or reducing depending on the particular sample size ratio. Therefore, with increasing $\delta$, $\delta =5$ gives higher coefficients of variation.

**Fig. 11:**
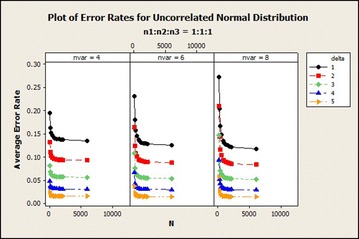
Average error rates of uncorrelated normal distribution for $\delta$: $n_1{:}n_2{:}n_3=1{:}1{:}1$

**Fig. 12:**
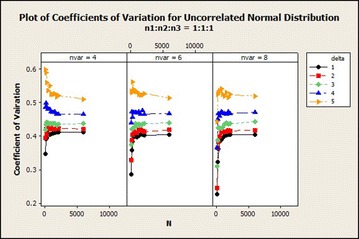
Coefficients of variation for uncorrelated normal distribution: $n_1{:}n_2{:}n_3=1{:}1{:}1$

From Fig. 11, we observed that the average error rates of the individual $\delta$s reduce as the sample size increases. There was about 3.19, 5.09, 6.81 % drop of the average error rate for $\delta =1$, variables 4, 6 and 8 respectively. The average error rates of $\delta =2$ for variables 4–6 exhibited about 2.00, 3.99, 6.65 % drop in the average error rates. In general, the average error rates decreased as $\delta$ increased irrespective of the number of variables and sample size ratios. The coefficient of variation of this distribution of sample size ratio 1 : 1 : 1 in Fig. 12 did not show any uniform pattern in the variabilities as $\delta$ increased but in general as $\delta$ increased, the variabilities also increased.

## Conclusion

The study focussed on the asymptotic performance of the QDF under correlated and uncorrelated normally distributed training samples. Under this distribution, the performance of the QDF under varying sampling ratios, selected number of variables and different group centroid separators were extensively studied. The QDF recorded minimum misclassification error rates and high variability as the sample size increased asymptotically under correlated normal distribution, thereby increasing the accuracy of classification of observations with the function. The performance of the QDF deteriorated when the sample size ratio was 1:2:3 as $\delta$ increased with increasing sample size. However, the performance of the function was appreciably good under both correlated and uncorrelated normal distributions when their estimated average misclassification error rate decreased with increasing number of variables (from 4 to 8). This results shows some partial conformity with the study of Lawoko (1988) where the researcher found that the efficiency of the QDF and other classifiers are generally lowered by positively correlated training observations. Generally, the study found that, the QDF performed better resulting in the reduction in misclassification error rates as group centroid separator increases with non increasing sample size and under correlated training samples. This results therefore shows some partial conformity with the studies by Marks in 1974. Marks approached the problem of discrimination by comparing the performance of QDF with other classifiers. Although he considered only two populations, the QDF performance was abysmal under small sample size selection when covariance matrices were nearly equal with large dimensions.
